# Effects of Substrate-Coating Materials on the Wound-Healing Process

**DOI:** 10.3390/ma12172775

**Published:** 2019-08-29

**Authors:** Jin-Young Lin, Kai-Yin Lo, Yung-Shin Sun

**Affiliations:** 1 Department of Physics, Fu-Jen Catholic University, New Taipei City 24205, Taiwan; 2 Department of Agricultural Chemistry, National Taiwan University, Taipei 10617, Taiwan

**Keywords:** wound-healing assay, cell migration, wound-healing rate, fibroblasts, surface coating

## Abstract

The wound-healing assay is commonly and widely used for investigating collective cell migration under various physical and chemical stimuli. Substrate-coating materials are shown to affect the wound-healing process in a cell-type dependent manner. However, experiment-to-experiment variations make it difficult to compare results from different assays. In this paper, a modified barrier wound-healing assay was reported for studying the wound-healing process on different substrates in one single petri dish. In short, half of a dish was covered with the tape, and coating materials, poly-l-lysine and gelatin, were applied to the surface. After peeling off the tape, half of the surface was coated with the desired material. Then a customized barrier was placed inside the dish to create the wound. The results indicated that surface coating did not affect cell proliferation/viability, and the wound-healing rate increased in coated surfaces compared to uncoated ones. The present study provides a platform for further understanding the mechanisms of substrate coating-dependent wound-healing processes.

## 1. Introduction

Cell migration is directed by various chemical and physical cues, phenomena named “-taxis”. Chemotaxis, the directional migration of somatic cells in response to certain chemical gradient, plays important roles in many physiological processes such as cancer metastasis [1,2] and cell development [3,4]. Electrotaxis, characterizing how adherent cells move under the stimulus of direct current or alternating current electric fields, has been reported to be crucial in wound healing [5,6] and nerve regeneration [7]. Other types of “-taxis” have been identified and investigated, including phototaxis (stimulated by a light gradient) [8], aerotaxis (stimulated by an oxygen gradient) [9], thermotaxis (stimulated by a temperature gradient) [10], durotaxis (stimulated by a stiffness gradient) [11], and magnetotaxis (stimulated by a magnetic field gradient) [12]. Induced by one or more of above-mentioned stimuli, cells can migrate collectively as a consequence of cell–cell communication and cell-environment interactions. Cells usually form the so-called self-assembled monolayers where they are attached to each other in biochemical and/or mechanical ways. To follow the dynamic process of collective cell migration as well as better understand its underlying mechanisms, researchers have developed various in vitro techniques, including wound-healing assays [13,14]. 

In a wound-healing assay, a cell-free region is created from a cell monolayer. Cells migrate to cover the wound, and time-lapse images are recorded using a microscope. Then based on these images, wound-healing rates can be calculated. The most common method for creating a wound is to scratch out a cell-free area using a tip or needle [15,16,17]. This scratch wound-healing assay has been commercialized as the CellPlayer Migration Assay by Essen BioScience (Ann Arbor, MI, USA) [18]. The advantages of this method include (1) it is easily conducted and quick, and (2) it can be applied on any substrates. However, using tips to mechanically create the wound could do damage to the cells and the cell culture surface. It is also difficult to control the size and shape of the wound from experiment to experiment. All these make the results unreliable and hard to be compared. Instead, by placing a stopper onto the substrate prior to seeding cells and waiting until a cell monolayer is formed, a cell-free region is created after removing the barrier [13,19]. In this barrier wound-healing assay, the cells and the surface remain intact, and the size and shape of the wound can be precisely controlled. Platypus Technologies (Madison, WI, USA) and Ibidi (Martinsried, Germany) have commercialized this assay by placing stoppers inside each well of a 96-well plate to increase the experimental throughput. As substitutes, various wound-healing assays have been developed, including those based on laser and ultraviolet light ablation [20,21], current-induced cell electroporation [22], and trypsin-caused cell dissociation in microfluidic devices [23,24].

The wound-healing process, quantified as the healing rate, is affected by a number of factors, such as cell type, cell culture substrate (coating and stiffness), and other physical /chemical stimuli. It was found that, using fibroblast cell line NIH 3T3 as the model, both cell culture serum and electric field increased the wound-healing rate obviously [5]. And in the same cell model, chemicals β-lapachone and honokiol were shown to increase and decrease the wound-healing rates, respectively [5,21]. As far as the cell culture surface is concerned, skin explants cultured on 2–50 kPa collagen-coated substrates rapidly re-epithelialized within 10–15 h, but in harder (1 GPa) and other coatings (tenascin, fibronectin, and laminin), the wound recovered slowly [25]. The wound-healing rate depends on not only the substrate-coating material but also on the cell type. Klein et al. reported that fibronectin accelerated the collective migration of Schwann cells, but collagen I, laminin, poly-l-lysine, and poly-l-ornithine showed little or no migration-promoting effects [26]. However, another research using prostate cancer cells as the model exhibited conflicting results. It was observed that cells grown in the presence of laminin migrated 62% faster than the control cells, but poly-l-lysine, poly-l-ornithine, and fibronectin caused cells to migrate slower than the control, displaying reduced wound-healing rates of 15%, 33%, and 20%, respectively [27]. In these two studies, the scratch wound-healing assays were used, which could damage the coatings and possibly cause further experiment-to-experiment variations. 

To get rid of the drawbacks of the scratching method, here we report a modified barrier wound-healing assay for investigating collective cell migration on different coated substrates. Fibroblasts are commonly used as the wound-healing model because they play critical roles in processes of breaking down the fibrin clot, creating new extra cellular matrix, and contracting the wound [28,29,30]. One half of a culture dish was coated with poly-l-lysine or gelatin, and the other half was uncoated. Prior to seeding cells, a barrier was placed onto the substrate. After a cell monolayer was formed, the stopper was removed to form a wound. The wound-healing processes under coated and uncoated surfaces were followed simultaneously to obtain the healing rates for comparison. This design greatly eliminated the possibilities of damages to the coatings and run-to-run variations. The present study provides a platform for accurate and unbiased investigation of substrate coating-dependent wound-healing processes.

## 2. Materials and Methods 

### 2.1. Cell Culture and Chemicals

The mouse embryonic fibroblast cell line NIH 3T3 was purchased from the Bioresource Collection and Research Center (BCRC), Hsinchu, Taiwan. A complete medium consisting of Dulbecco’s modified eagle medium (DMEM, Gibco, Waltham, MA, USA) and 10% calf serum (CS, Invitrogen, Carlsbad, CA, USA) was used for cell culture. The cells were incubated in tissue culture polystyrene flasks (Corning, Corning, NY, USA) in 5% CO_2_ at 37 °C until a density of 5~6 × 10^4^ cells/cm^2^ was reached. For surface coating, gelatin (Sigma, St. Louis, MO, USA) and poly-l-lysine (Sigma, St. Louis, MO, USA) were diluted in 1× phosphate-buffered saline (PBS) into concentrations of 0.1% (*w*/*w*) and 0.1 μg/mL, respectively, as recommended by the manufacturer. Coomassie Brilliant Blue (Bionovas, Toronto, ON, Canada) was diluted into a concentration of 2.5 g/L (in 0.1 L acetic acid, 0.3 L methanol, and 0.6 L ultrapure water) for verifying the presence of gelatin and poly-l-lysine.

### 2.2. Cell Viability Assay

The proliferation and viability of fibroblasts were quantified using the MTT (3-(4,5-Dimethylthiazol-2-yl)-2,5-diphenyltetrazolium bromide) assay (Sigma). This yellow tetrazolium salt was transformed into purple formazan crystal by live cells. Fibroblasts were passaged to a 12-well plate divided into three groups: Control (uncoated), PLL (coated with 0.1 μg/mL poly-l-lysine), and Gelatin (coated with 0.1% gelatin). After 24 h, 400 μL of MTT solution (0.5 mg/ml in DMEM) was added to each well for incubation at 37 °C for 2 h. Then 400 μL of solubilization solution, dimethyl sulfoxide (DMSO), was added to the wells for 5 min incubation. The absorbance was measured using an ELISA (enzyme-linked immunosorbent assay) plate reader (Tecan, Männedorf, Switzerland) at 570 nm. Two independent experiments were performed on separate plates.

### 2.3. Fabrication of Barrier

**Figure 1:**
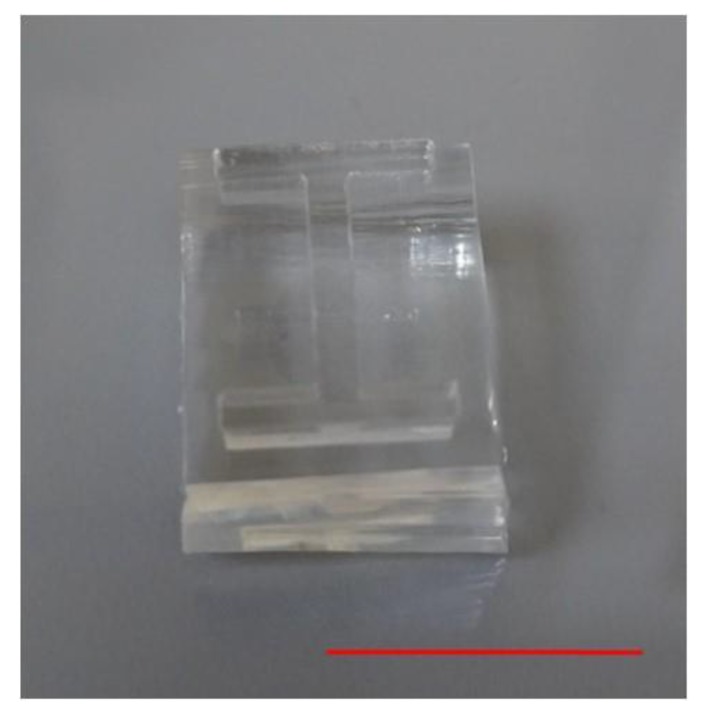
Picture of the customized PDMS I-shaped barrier. Scale bar = 1.5 cm.

The I-shaped barrier was made of polydimethylsiloxane (PDMS, Dow Corning Sylgard 184, Midland, MI, USA). First, a negative polymethylmethacrylate (PMMA) mold was fabricated using a CO_2_ laser scriber (MS640D, Ming-Cheng Technics Corp., Nantou, Taiwan). A number of these molds were put inside a petri dish (diameter = 10 cm, SPL Life Sciences, Gyeonggi-do, Korea), and the PDMS solution (1/10, *w*/*w* curing agent to prepolymer) was poured into the dish to cover the molds. The molds were put under vacuum (~60 Torr) for 30 min and then baked at 50 °C for 4 h and then left under sterile conditions overnight. The I-shaped PDMS barriers were cut off and removed from the dish in preparation for use in experiments (see Figure 1).

### 2.4. Experimental Procedure

**Figure 2:**
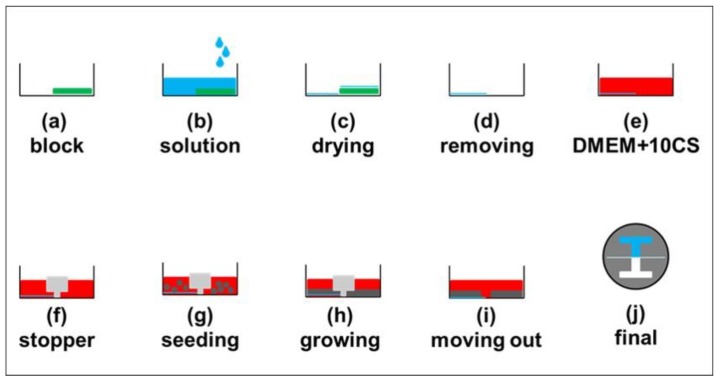
Experimental procedure. (**a**–**i**) Side view of the dish. (**a**) Block half of the dish with the tape. (**b**) Pour in the solution. (**c**) Wait until dried. (**d**) Remove the tape. (**e**) Add in the culture medium. (**f**) Place the stopper in the middle of the dish. (**g**) Seed in cells. (**h**) Wait until a cell monolayer was formed. (**i**) Remove the stopper. (**j**) Top view of the dish. Green: tape. Blue: washing buffer (PBS). Red: Cell medium (DMEM + 10% CS). Light gray: stopper. Dark gray: cell.

The experimental procedure is illustrated in Figure 2 and described as follows. First, half of a petri dish (diameter = 3.5 cm, TrueLine, Rochester, NY, USA) was blocked with the tape (thickness = 60 μm, 8018, 3 M) (Figure 2a). The solution, washing buffer (PBS, for Control, Sigma, St. Louis, MO, USA), 0.1% gelatin, or 0.1 μg/mL poly-l-lysine, was poured into the dish (Figure 2b). After 30 min, the solution was removed, and the dish was washed with PBS a few times and then dried (Figure 2c). The tape was removed (Figure 2d) and cell medium (DMEM + 10% CS) was poured into the dish (Figure 2e). The I-shaped barrier was placed in the middle of the dish (Figure 2f) and then 2.5 × 10^5^ cells were seeded inside (Figure 2g). After a cell monolayer was grown (Figure 2h), the barrier was removed (Figure 2i). Figure 2j shows the top view of the dish, where the upper part was coated (blue), and the lower part was uncoated (white).

### 2.5. Data Analysis

**Figure 3:**
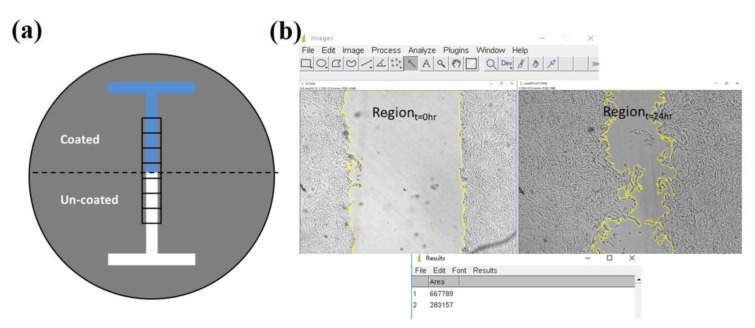
(**a**) Seven images were taken at one time point. The upper three images were used for the coated substrate. The lower three images were used for the uncoated substrate. (**b**) ImageJ was used to draw the boundaries and calculate the area of the wound.

The wound-healing process was recorded using a bright-field inverted microscope (ESPA, Hsinchu, Taiwan). Seven images (see Figure 3a) were taken at 0, 12, 24, 36, and 48 h after removing the barrier. They were further analyzed using ImageJ, which is a free Java-based software developed by the National Institutes of Health (Version 1.50c, NIH, Bethesda, MD, USA). As shown in Figure 3b, this software was used to draw the boundaries of the wound at different time points. The area enclosed by these boundaries could be calculated. And the wound-healing rate is calculated as $\frac{\left(R_{i}-R_{f}\right)}{R_{i}}\times 100\%,$ where *R_i_* and *R_f_* are the initial and final areas of the wound, respectively. For each condition, three independent runs were performed. Therefore, there were a total of nine (three images in each of three runs) data points to be analyzed to get the standard error of the mean (SEM).

## 3. Results and Discussion

### 3.1. Verifying the Presence of Coating Materials

**Figure 4:**
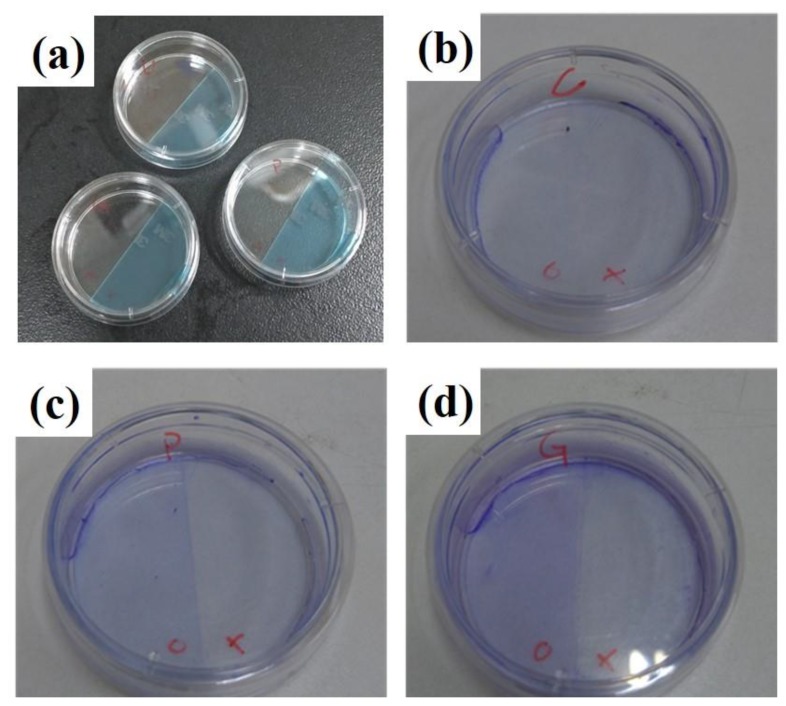
(**a**) Half of the petri dishes were blocked with the tapes. Pictures of Coomassie Brilliant Blue staining of (**b**) phosphate-buffered saline (PBS)-coated (C), (**c**) poly-l-lysine-coated (P), and (**d**) gelatin-coated dishes (G). Circle: coated half. Cross: uncoated half.

Coomassie Brilliant Blue is commonly and widely used for staining proteins. After blocking half of the petri dishes with the tapes (see Figure 4a), solutions of PBS (C, for control), poly-lysine (P), and gelatin (G) were added into the dishes. Then solutions were removed, the dishes were washed with PBS, and the tapes were removed. Stained with Coomassie Brilliant Blue for 30 min and washed with ultrapure water a few times, the dishes were photographed and are shown in Figure 4b–d. As clearly seen, surfaces coated with poly-l-lysine (Figure 4c left, marked with a circle) and gelatin (Figure 4d left, marked with a circle) were much bluer than those that were uncoated (Figure 4b–d right, marked with crosses) and coated with PBS (Figure 4b left, marked with a circle). Therefore, it was verified that poly-l-lysine and gelatin were successfully coated on the surfaces. The concentrations used here were recommended by the manufacturer for surface modification. Different dilutions were also tried, but no better results were obtained (data not shown).

### 3.2. Cell Morphology and Proliferation/Viability

**Figure 5:**
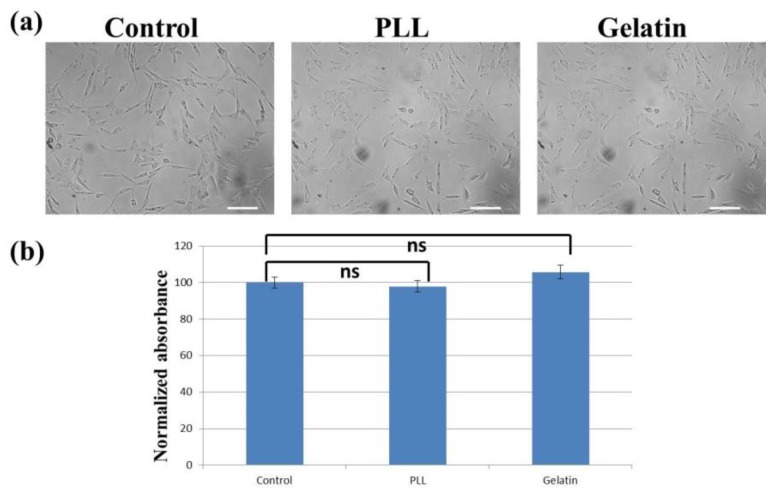
(**a**) Cells grown on uncoated (left), poly-l-lysine-coated (middle), and gelatin-coated (right) surfaces after 24 h. Scale bar = 100 μm. (**b**) Cell proliferation/viability on different surfaces after 24 h. Control: uncoated. PLL: Poly-l-lysine. Statistical analysis was performed on eight independent data points (see Section 2.2). Student’s *t*-tests were performed. ns: no statistically significant difference (*p* > 0.05).

Figure 5a shows the images of cells grown on different substrates after 24 h. Without detailed analysis, the sizes and shapes of cells on uncoated and coated surfaces were similar, suggesting that these coatings did not alter cell morphology. The result of the 24 h MTT assay is shown in Figure 5b, where the absorbance was normalized to that of the control (uncoated) group. As indicated, the normalized values were 97.9% and 105.7% for poly-l-lysine- and gelatin-coated surfaces, respectively. Compared with the uncoated surface, these two coatings slightly reduce and increase the proliferation/viability of fibroblasts, respectively. Student’s *t*-tests were performed, showing no statistically significant differences. Therefore, it was suggested that these two materials did not significantly affect cell proliferation/viability. It was reported that Schwann cells cultivated on poly-l-lysine showed no difference in cell viability when compared with the cells cultured on uncoated polystyrene after two days [26]. Human colon adenocarcinoma cells also exhibited similar proliferation rates on gelatin-coated and uncoated microfluidic channels after two days of culture [31].

### 3.3. Dependence of Substrate-Coating Materials on the Wound-Healing Process

**Figure 6:**
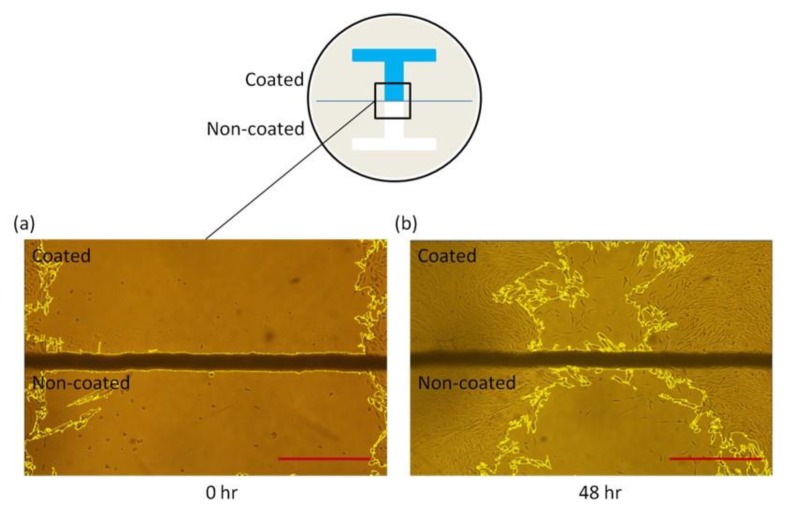
A representative image showing the wound-healing process in gelatin-coated and uncoated surfaces. (**a**) The wound at time = 0 h. (**b**) The wound at time = 48 h. Scale bar = 500 μm.

**Figure 7:**
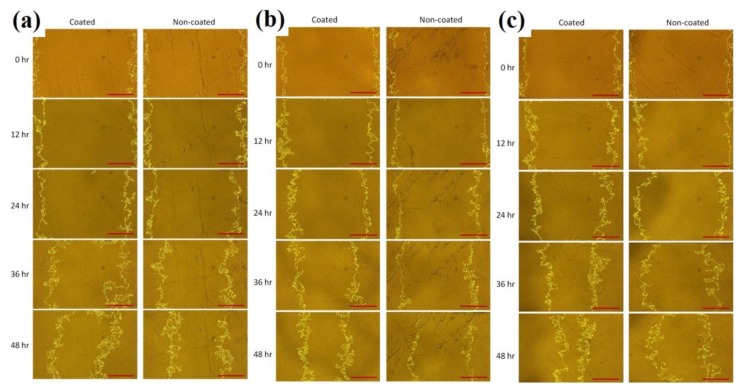
Images of the wounds at different time points after removing the barrier. (**a**) PBS-coated and uncoated surfaces. (**b**) Poly-l-lysine-coated and uncoated surfaces. (**c**) Gelatin-coated and uncoated surfaces. Scale bar = 500 μm.

**Figure 8:**
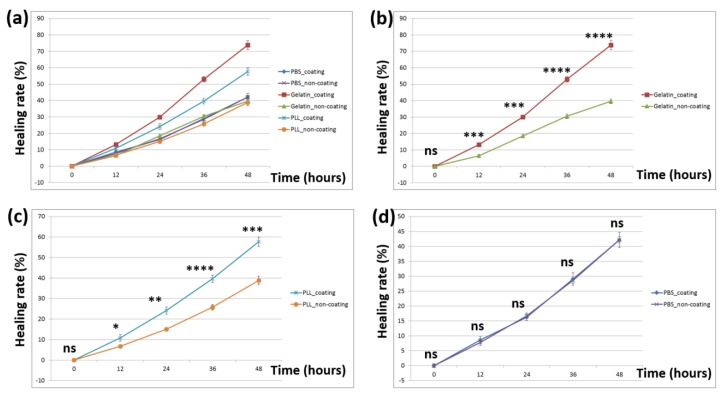
(**a**) Calculated wound-healing rates under all conditions. Statistical analysis was performed on nine independent data points (see Section 2.5). Student’s *t*-tests were performed between (**b**) gelatin-coated and uncoated surfaces, (**c**) poly-l-lysine-coated and uncoated surfaces, and (**d**) PBS-coated and uncoated surfaces. ns: no statistically significant difference (*p* > 0.05); *: *p* < 0.05; **: *p* < 0.01; ***: *p* < 0.001; ****: *p* < 0.0001.

The wound-healing process was followed by taking time-lapse images at 0, 12, 24, 36, and 48 h after removing the barrier. Figure 6 shows a representative image taken in the boundary between gelatin-coated and uncoated surfaces. The width of the wound was around 1.5 mm, equal to that of the I-shaped barrier. After 48 h, the wound in the coated surface was nearly closed compared to that in the uncoated surface. Figure 7 shows the images of the wounds at different time points. As obviously indicated, cells migrated faster on poly-l-lysine- (Figure 7b Coated) and gelatin-coated (Figure 7c Coated) surfaces than on PBS-coated (Figure 7a Coated) and uncoated surfaces (Figure 7a–c) Non-coated). There were two types of control conditions. In one, half of the dish was blocked, meaning that this part was uncoated. In the other, PBS was used as the coating material, suggesting that the entire dish was uncoated. Comparing the coated and non-coated images in Figure 7a, it was implied that the residues after removing the tapes (if there were any) did not affect the wound-healing process. Quantitatively, the wounding areas at different time points were measured and the corresponding wound-healing rates were calculated and shown in Figure 8a. Student’s *t*-tests were performed on all time points. Both gelatin- and poly-l-lysine-coated surfaces accelerated the wound-healing processes compared with uncoated ones. For gelatin, the healing rates were 74%, 53%, 30%, and 13% at 48 h, 36 h, 24 h, and 12 h, respectively. These values were 89%, 60%, 57%, and 32% higher than those of uncoated surfaces. As shown in Figure 8b, the *p*-value between gelatin-coated and uncoated groups at 48 h was less than 0.0001. For poly-l-lysine, the healing rates were 58%, 40%, 24%, and 11% at 48 h, 36 h, 24 h, and 12 h, respectively. Similarly, these values were 49%, 54%, 60%, and 57% higher than those of uncoated surfaces. As shown in Figure 8c, the *p*-value between poly-l-lysine-coated and uncoated groups at 48 h was less than 0.001. In the control (PBS) group, the healing rates in the PBS-coated surface were close to those in the uncoated surface, with errors of 0.24%, 1.7%, 3%, and 10% at 48 h, 36 h, 24 h, and 12 h, respectively. The *p*-value between these two groups indicated no statistically significant difference as shown in Figure 8d. This again verified that the tape did not affect the wound-healing rate.

Gelatin, an irreversibly hydrolyzed form of collagen, is widely used as a coating material in cell culture for improving cell attachment [32]. It was reported that smooth muscle cells migrated faster on the gelatin-coated surface than on the uncoated polystyrene surface [33]. An increase of 63% in the migration rate from 24 to 48 h was observed in a barrier wound-healing assay [33]. Under the same conditions, for human umbilical vein endothelial cells, the migration rate was even six times faster on the gelatin-coated surface than on the uncoated polystyrene one [33]. Poly-l-lysine is a synthetic, positively charged amino acid chain commonly used as a coating material to enhance cell adhesion [34,35]. In the present study, poly-l-lysine was shown to accelerate wound healing in fibroblasts. Somaiah et al. found that, by using the scratching method, mesenchymal stem cells migrated faster on the poly-l-lysine surface (with an average migration speed of 6 μm/h) than on the uncoated one (with an average migration speed of 3.6 μm/h) [36]. However, contrary results were reported on different cell lines. In a scratch wound-healing assay using Schwann cells as the model, the wounding area after 48 h was still 67.5% of the original one on the poly-l-lysine coated surface, compared to 54.4% on the uncoated surface [26]. Results from another scratch assay indicated that poly-l-lysine caused prostate cancer cells to migrate slower than the control, displaying a reduction of wound density by 15% [27]. It is therefore suggested that the effect of poly-l-lysine coating on the wound-healing process is cell type-dependent.

## 4. Conclusions

In this study, we reported a modified barrier assay for studying the effects of substrate-coating materials on the wound-healing process. The migration of fibroblasts on both coated and uncoated surfaces was followed simultaneously in one single petri dish. This greatly eliminated the experimental run-to-run variations. The results indicated that (1) both poly-l-lysine and gelatin coatings had almost no effects on cell proliferation and viability, and (2) compared to the uncoated one, cells migrated faster on coated surfaces by showing increased wound-healing rates. We will be working on investigating the effects of other coating materials such as collagen, fibronectin, laminin, and poly-l-ornithine. The present platform is of help in further understanding the mechanisms of substrate coating-dependent wound-healing processes.
