# Correction: Lin. et al. Effects of Substrate-Coating Materials on the Wound-Healing Process. *Materials* 2019, *12*, 2775

**DOI:** 10.3390/ma13010248

**Published:** 2020-01-06

**Authors:** Jin-Young Lin, Kai-Yin Lo, Yung-Shin Sun

**Affiliations:** 1 Department of Physics, Fu-Jen Catholic University, New Taipei City 24205, Taiwan; aasd59472@gmail.com; 2 Department of Agricultural Chemistry, National Taiwan University, Taipei 10617, Taiwan; kaiyin@ntu.edu.tw

The authors wish to make the following correction to this paper [1]. Due to mislabeling, replace:

**Figure 5:**
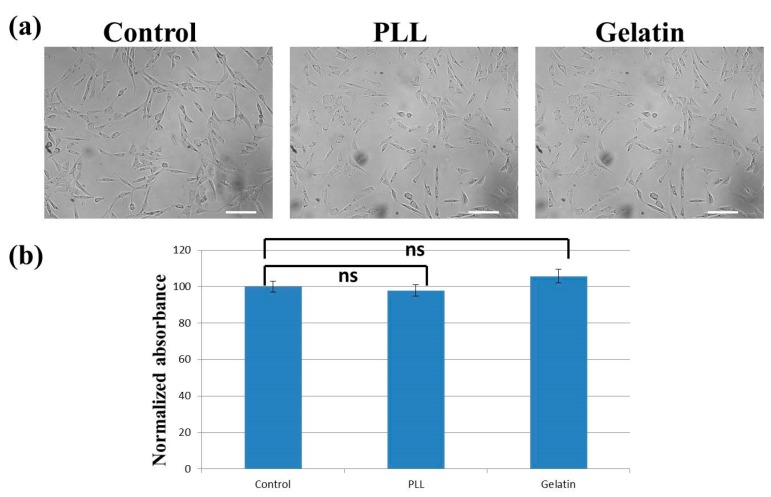
(**a**) Cells grown on uncoated (**left**), poly-L-lysine-coated (**middle**), and gelatin-coated (**right**) surfaces after 24 h. Scale bar = 100 μm. (**b**) Cell proliferation/viability on different surfaces after 24 h. Control: uncoated. PLL: Poly-L-lysine. Statistical analysis was performed on eight independent data points (see Section 2.2). Student’s t-tests were performed. Ns: no statistically significant difference (*p* > 0.05).

with

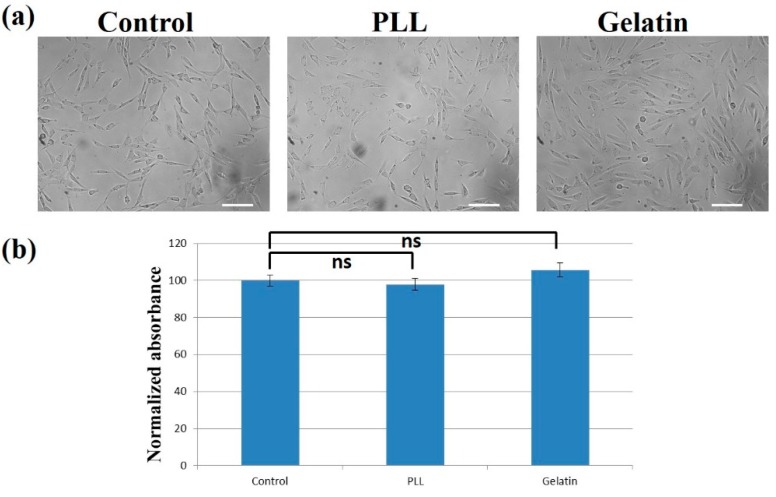



These changes have no material impact on the conclusions of the paper. The authors would like to apologize for any inconvenience caused to the readers by these changes.
